# Using a Simple Neural Network to Delineate Some Principles of Distributed Economic Choice

**DOI:** 10.3389/fncom.2018.00022

**Published:** 2018-03-28

**Authors:** Pragathi P. Balasubramani, Rubén Moreno-Bote, Benjamin Y. Hayden

**Affiliations:** ^1^Brain and Cognitive Sciences, Center for Visual Science, Center for the Origins of Cognition, University of Rochester, Rochester, NY, United States; ^2^Department of Information and Communications Technologies, Center for Brain and Cognition, University Pompeu Fabra, Barcelona, Spain; ^3^Serra Húnter Fellow Programme, University Pompeu Fabra, Barcelona, Spain; ^4^Neuroscience and Center for Magnetic Resonance Research, University of Minnesota, Minnesota, MN, United States

**Keywords:** neuroeconomics, distributed network, neural network, modular network, parallel distributed system

## Abstract

The brain uses a mixture of distributed and modular organization to perform computations and generate appropriate actions. While the principles under which the brain might perform computations using modular systems have been more amenable to modeling, the principles by which the brain might make choices using distributed principles have not been explored. Our goal in this perspective is to delineate some of those distributed principles using a neural network method and use its results as a lens through which to reconsider some previously published neurophysiological data. To allow for direct comparison with our own data, we trained the neural network to perform binary risky choices. We find that value correlates are ubiquitous and are always accompanied by non-value information, including spatial information (i.e., no pure value signals). Evaluation, comparison, and selection were not distinct processes; indeed, value signals even in the earliest stages contributed directly, albeit weakly, to action selection. There was no place, other than at the level of action selection, at which dimensions were fully integrated. No units were specialized for specific offers; rather, all units encoded the values of both offers in an anti-correlated format, thus contributing to comparison. Individual network layers corresponded to stages in a continuous rotation from input to output space rather than to functionally distinct modules. While our network is likely to not be a direct reflection of brain processes, we propose that these principles should serve as hypotheses to be tested and evaluated for future studies.

## Introduction

Many distributed decision-making systems, such as honeybee swarms, democracies, and slime molds can select options based on their economic values (Bartels, 1988; Franks et al., 2002; Cohen et al., 2008; Seeley, 2010). Such systems are faced with different constraints from more conventional serial, localized, and modular systems. In modular decision-making systems, different elements of choice are handled by dedicated subsystems that are functionally separated and that have highly specialized roles. For example, in the brain, different components of decision-making, from evaluation to action selection, are sometimes proposed to be controlled by distinct brain structures (Hare et al., 2009, 2011; Padoa-Schioppa, 2011; Levy and Glimcher, 2012). In distributed systems, by contrast, individual elements follow similar repertoires, typically have more autonomy, and generally have limited and often stochastic ability to communicate with other units (Seeley and Buhrman, 1999; Couzin, 2009; Marshall et al., 2009; Mitchell, 2009; Eisenreich et al., 2017; Hunt and Hayden, 2017).

These two classes of systems may make choices based on distinct sets of principles. The brain is, of course, a complex organ with mixed distributed and modular elements. Nonetheless, many proposed models are far on the modular side of the continuum; that is, they envision that circuits corresponding to elements of choice are anatomically separate, either at the level of the brain region or the single neuron. Thus, for example, one region or circuit may evaluate offers, another may compare them, and a third may bind the results of the comparison with actions to produce an overt choice (e.g., Wong and Wang, 2006; Hare et al., 2009, 2011; Padoa-Schioppa, 2011; Levy and Glimcher, 2012; Ebitz and Hayden, 2016). Generally, in modular systems, value is computed and represented explicitly and in an abstract and universal format, often called a common currency (Landreth and Bickle, 2008; Chib et al., 2009; Padoa-Schioppa, 2011; Levy and Glimcher, 2012; Sescousse et al., 2015). Brain regions can be classified as pre- or post-decisional, based on whether they precede or follow the calculation of a common currency value. And value comparison can be said to take place in a particular space, typically in goods space or action space (e.g., Rangel et al., 2008; Levy and Glimcher, 2012; Bartra et al., 2013; Clithero and Rangel, 2013). Modular approaches often focus on the question of what is the single site in the brain at which comparison occurs, rather than how comparison reflects the interaction of qualitatively different elements (e.g., O'Doherty, 2004; Hare et al., 2008, 2011; Padoa-Schioppa and Assad, 2008; Kennerley et al., 2011; Padoa-Schioppa, 2011; Hauser et al., 2015). They also tend to explore how values are bound to actions (e.g., Padoa-Schioppa et al., 2006; Cravo et al., 2011; Hare et al., 2011; Padoa-Schioppa, 2011).

Surprisingly, most thinking on economic choices and their implementation in models has focused on modular networks where the division of labor is rather strict, in the sense that, for instance, one part of the network is devoted to value estimation while other part of the network is devoted to choice selection (Soltani et al., 2006; Soltani and Wang, 2010; Hunt et al., 2012; Chau et al., 2014; Rustichini and Padoa-Schioppa, 2015). Such work also parallels related perceptual decision-making models in which one part of the network performs feature or stimulus estimation separately and then send this information out downstream for the formation of a decision (Shadlen et al., 1996; Wang, 2002; Usher and McClelland, 2004; Moreno-Bote et al., 2007; Beck et al., 2008). The above work has provided a solid pillar to understand the neuronal mechanism under which economic choices might operate, especially because they model explicitly the time evolution of choice selection through detailed and biophysically plausible synaptic dynamics. Despite their large success, the principles under which economic choices operate under distributed systems have not been explored.

The goals of this perspective are to delineate the properties of distributed economic decision-making as a guide for future critical tests. Specifically, our first goal is to provide a proof-of-concept argument that a modular system is not *a priori* necessarily true, nor are ideas like common currency value representations, an abstract goods space, or a labeled line correspondence between specific neurons and offers. Our second goal is to delineate the properties of one example system, and use these as a lens through which to re-examine neural signals. We conclude that the field's strong focus on modular, as opposed to distributed, models of economic choice is premature.

**Figure 1:**
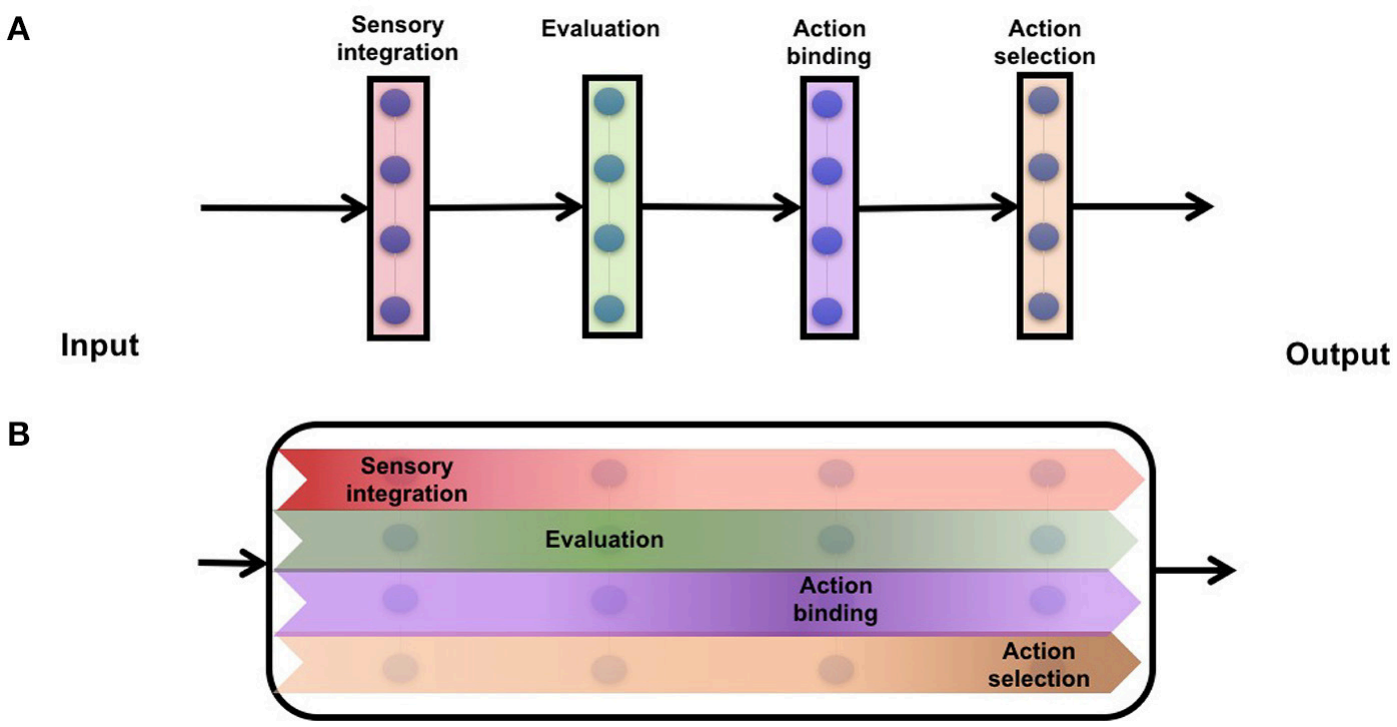
Cartoon schematic of information transforming from input to output spaces. **(A)** modular networks which encapsulate every component of decision making into discrete set of neurons **(B)** distributed networks which continuously rotates information from input to a decision.

As a guide to the basic properties of distributed choice systems we use a feed-forward neural network, similar to one that has been successfully used for vision (Orhan and Ma, 2017), to perform choices between two gambles (the specific gambling problem we chose is based on our own previous studies of such tasks; Strait and Hayden, 2013; Strait et al., 2014; Blanchard et al., 2015a; see also Blanchard et al., 2014, 2015b). We and others previously proposed that choice processes may be distributed. However, in the past we did not go, at a detailed level, into how that might occur. Here, we implement a neural network as the next small step in generating an intuition for how distributed choice processes may occur in the brain. The network is not designed to provide a realistic model of neural computations underlying choice; the real brain has many important features not included in our model (like its size, plasticity, feedback, oscillatory signals, etc.). Instead, our goal is to understand, in an abstract way, the general properties of a very simple distributed network that can solve the problems of economic choice (Figure 1).

## A neural network is a distributed choice system

As a lens through which to examine the mechanisms of economic choice, we consider a simple distributed chooser, a feed-forward network (Werbos, 1974; Minsky and Papert, 1988) that can perform a neuroeconomic task involving choice between two gambles. (We focused on gambles because they are mathematically well understood and yet not so simple as to be trivial, and because we have ready access to neural data in similar tasks).

Each of the two gambles (indicated by *i* = 1,2, for right and left spatial location) is described by a reward magnitude, *r*_*i*_, and the probability, *p*_*i*_, of obtaining it (the probability of not obtaining reward as outcome is the complementary probability 1−*p*_*i*_). The reward magnitude per gamble is drawn randomly and independently across trials, following a uniform discrete distribution in the interval [0,2] in steps of 0.2 (arbitrary units, a.u.). The probabilities are also drawn randomly across gambles and trials from a uniform discrete distribution in the interval [0,1] in steps of 0.1. Accordingly, the expected values (*EV*) for the two gambles varied randomly, defined as the product of the corresponding probabilities and rewards, that is, *EV*_*i*_ = *p*_*i*_*r*_*i*_.

We used a network consisting of 3 hidden layers (20 units in each hidden layer), 1 input layer and 1 output layer. The input layer contains 4 neurons (nodes), and the output consists of 2 neurons. The details of the network, such as number of layers or number of neurons are not critical, and the qualitative results discussed in this study apply to a large set of configurations. This supports the idea that the principles we designate here are rather general.

The rewards and probabilities of the gambles spatially presented on the right and left side of the screen define the input vector, ***I***, which correspond to the activity of the 4 neurons in the input layer,

(1) $$\begin{array}{r} \begin{array}{c} \text{I}=\left({r_{R},p}_{R},r_{L},p_{L}\right) \end{array} \end{array}$$

where the *i*-th entry of the input vector corresponds to activity of the *i*-th neuron in the input layer (*i* = 1,…,4). The gamble chosen by the network is read out from the two-neuron output layer (each neuron described by label *i* = 1,2) by determining which of the two neurons have the largest activity. That is, if neuron, *i* = 1 has larger activity than neuron, *i* = 2, then the gamble on the right is chosen, while the gamble on the left is chosen in the converse configuration.

To train our network we used several versions of the backpropagation algorithm (Rumelhart et al., 1986; Hoskins and Himmelblau, 1992; Boyan and Moore, 1995) to test the generality of our results, finding always very similar results across algorithms, and also across different realizations of the initial configurations of the network. For concreteness, we only report the results of the following algorithm. We take the subscripts (*n, i*) to represent neuron *i* in layer *n*. The activity (e.g., firing rate) of such a neuron is denoted *y*_*n, i*_. The number of hidden layers goes from 1 to *N*, and the number of neurons in each layer are 20, *i* = {1, …, 20}, except for the output layer (*n* = *N*), for which there are only two neurons *i* = {1, 2}. Note that the input layer, consisting of four neurons, taking values as in Equation (1), is considered to be layer *n* = 0.

At any trial to the network is presented with a choice between two gambles, described by the random vector in Equation (1). The gamble chosen corresponds to the neuron with the highest activity in the output layer, with activity:

(2) $$\begin{array}{r} \begin{array}{c} y_{chosen}^{N}=\max\left(y_{1}^{N},y_{2}^{N}\right) \end{array} \end{array}$$

In case that the two neurons have the same activity, one of them is chosen randomly. The goal of the network is to minimize the difference between the chosen activity and the obtained reward, *R*_*t*_, over trials

(3) $$\begin{array}{r} \begin{array}{c} E=\frac{1}{2}\sum_{t}e_{t}^{2}=\frac{1}{2}\sum_{t}{\left(R_{t}-y_{chosen,t}^{N}\right)}^{2}\; \end{array} \end{array}$$

We take gradient descent over this cost function by changing the weights of the network locally and incrementally over trials, using the back-propagation algorithm (Werbos, 1974; Rumelhart et al., 1986; Haykin and Network, 2004). The following describes the general algorithm for a given input at a trial *t*, and hence the subscript *t* is omitted for all equations from now on.

Denoting the weights connecting layers *n* and *n*-1 by $w_{ij}^{n}$, then the activity in one layer as a function of the previous layer is written as

(4) $$\begin{array}{r} \begin{array}{c} y_{i}^{n}=f\left(\sum_{j}w_{ij}^{n}\;y_{j}^{n-1}\right)\;, \end{array} \end{array}$$

where *f* is the neuron's activation function, taken to be a hyperbolic tangent with slope parameter equal to 3, *f*(*x*) = tanh(3*x*), (this choice leads to an initial good scaling of inputs into the first layer). The thresholds or biases (Rumelhart et al., 1986; Haykin and Network, 2004) are set to zero. We generalize Equation (4) for the special case of the first layer, *n* = 1, by taking $y_{j}^{0}=I_{j}$, that is, the activities of the input layer, Equation (1). The above equations define the activity of any neuron of the network at any given trial, with gambles as defined by the input vector, Equation (1).

Because the activity of the neuron associated to the chosen gamble in trial is the one that tries to predict the outcome, the input weights of the neuron associated with the chosen gamble in any single trial are updated, while the input weights of the neuron associated with the unchosen gamble are left constant in that trial. We denote by $\delta_{i}^{n}$ in every trial the error associated to neuron (*n, i*) in the network. These errors take the values in Equation (5) for the output layer,

(5) $$\begin{array}{r} \begin{array}{c} \delta_{i}^{N}=E_{i}\times \;f^{\prime }\left(\sum_{j}w_{ij}^{N}\;y_{j}^{N-1}\right) \end{array} \end{array}$$

and the following values

(6) $$\begin{array}{r} \begin{array}{c} \delta_{j}^{n}=\underset{i}{\sum }\delta_{i}^{n+1}w_{ij}^{n+1}\;f^{\prime }\left(\sum_{k}w_{jk}^{n}\;y_{k}^{n-1}\right)\; \end{array} \end{array}$$

for any other layer below it (*n* < *N*), where *f* ' is the derivative of the activation function (in our case, *f*′(*x*) = 1−*tanh*6*x*). Finally, the per trial update equation the weights per trial can be simply expressed as

(7) $$\begin{array}{r} \begin{array}{c} \Delta w_{ij}^{n}=\eta \;\delta_{i}^{n}{\;y}_{j}^{n-1}\;, \end{array} \end{array}$$

where η is the learning rate (η = 0.01). To keep the weights between bounds, at every trial after applying Eq. (6) we normalized all them by the maximum value of $w_{ij}^{n}$ across all neurons in layer, *n*, denoted $w_{\max}^{n}$, by

(8) $$\begin{array}{r} \begin{array}{c} w_{ij}^{n}\to \frac{w_{ij}^{n}}{w_{\max}^{n}}\; \end{array} \end{array}$$

We also used other versions of the background algorithm without weight normalization, giving very similar results to the ones shown here.

The parameters of the network are updated trial-by-trial using Equation (7), and the performance is tracked for a total of 3,000 trials. The responses of neurons in the final 1,000 trials portraying stationary performance through a plateau are used for analysis. The final results are an average of 300 networks that differ in their initial values, randomly and i.i.d. following a uniform distribution in the interval [−0.01, 0.01]. Thus, our results are not specific to any particular initial configuration of network connectivity.

The performance of the network is measured by its ability to choose the best of the offers. As the outcome is probabilistic, the network cannot fully predict and learn the actual outcomes beyond the average quantities. We expect, however, that the network as a whole is able to learn the abstract notion of ‘expected value’ from the observations and range of outcomes.

Correlations between neural responses to various task parameters were performed using Pearson linear correlations, and regressions were performed using linear regressions with single predictor. Tuning or coding of a neuron is defined when its activity significantly (*p* < 0.05) correlates with task variables such as offer value, probability of offer. There is a possible confound of the extent of training on the computed *p*-value. To get around the confound, we limit network learning to the saturation by following the criterion limit of performance greater than 80% in a total of 3,000 trials, a percentage similar to that reported during experimental training of humans and monkeys by our earlier studies. Also, we particularly focus on the differences in neuronal activity across layers, and not on the magnitude of individual neuronal responses. Because training was the same across layers/conditions, the relative values are (somewhat) meaningful, even if the absolute values are not. They help us to discuss the significance of differences in encoding of variables such as offer reward magnitude, offer probability, by neurons across layers. We compare them to neurophysiological data from various brain structures in the light of distributed coding.

Some variables of interest are offer reward magnitude (rew), offer probability (prob), both of which can be directly taken from the network input. Others are spatial position of offers (pos), and choice. The choice is a categorical variable computed based on the maximum of network output as described in the Methods. Spatial information, i.e., the encoding of offer side, is computed by finding the response activities to the same offer (reward and probability) when it appeared in position 1 (offer side is left, first two input terms) vs. position 2 (offer side is right, third and fourth term), and regressing the responses against their positions (1 vs. 2), irrespective of the other offer.

To analyze lesions, the activities of a proportion of neurons [5, 10, 25, 50, 75%] that are randomly selected in a given layer are turned to zero, after network stabilizes at trial = 3,000. The lesioned net is run for 1,000 trials and for 300 realizations of the initial conditions, with no additional training, for further analysis.

## Basic behavior of the network

**Figure 2:**
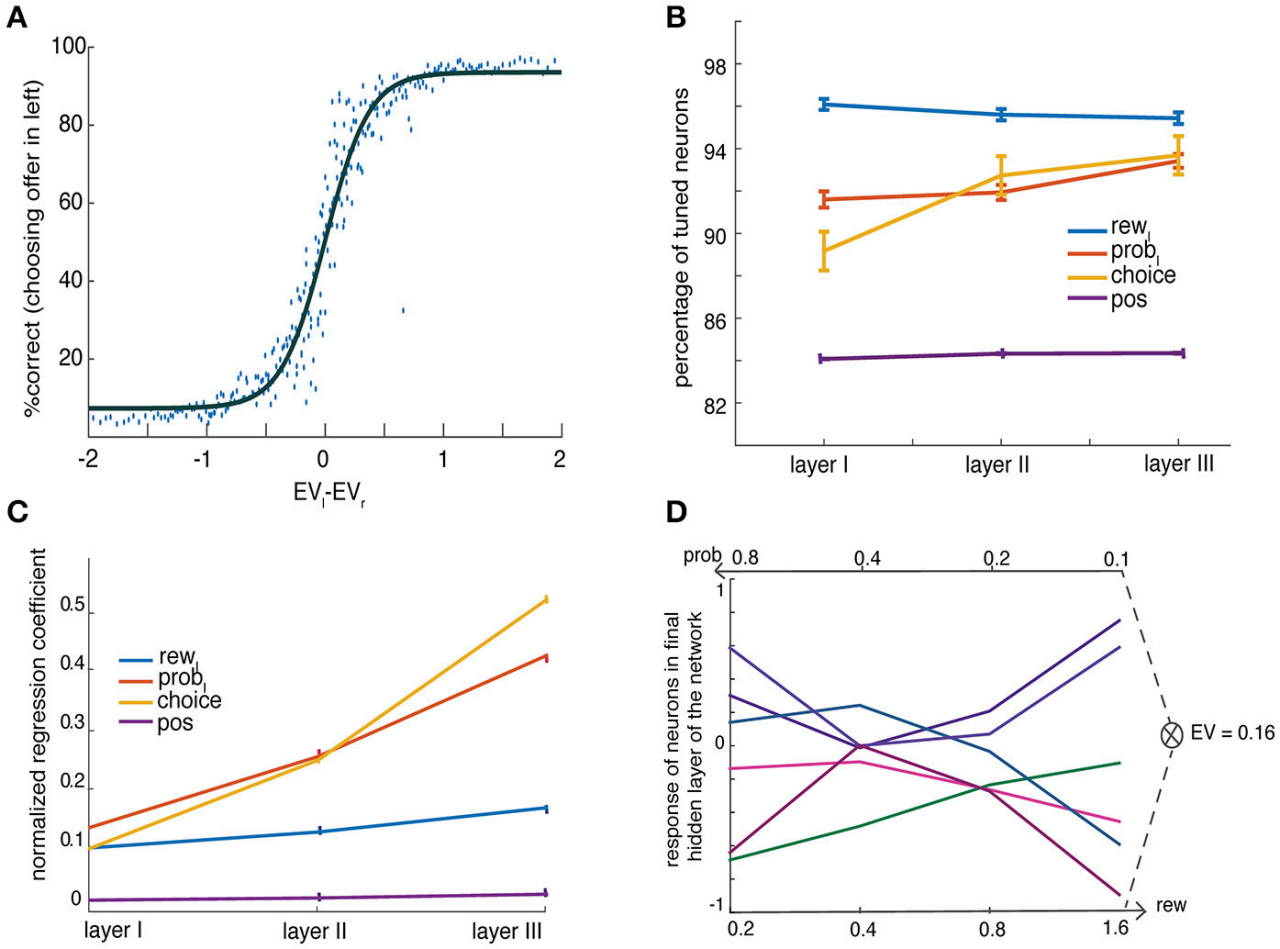
Basic behavior of the network. **(A)** Percent choosing the offer presented in left as a function of value difference between left and right offers (shaded in gray) shows a characteristic sigmoidal curve fit (averaged over *N* = 300 network instances) **(B)** Percent tuning for task parameters show ubiquitous encoding of them throughout the network. The panel shows percentage encoding of offer (left) value, *rew*, offer (left) probability, *prob*, choice and offer's spatial position, *pos*, in three layers of the network **(C)** Regression coefficients (β) of a linear regression model with a single regressor show increase in encoding of task parameters with increase in levels of layers. The figure shows the variance of observed regression coefficients for offer value (rew, left), offer probability (prob, left), choice and offer's spatial position (pos), for neurons across three layers. The spatial information is computed as the difference in responses to offer side for the same offer magnitude as described in the Methods. Error bars denote SEM **(D)** No common currency is found. The activity of several neurons in the third layer is shown as a function of reward and probability while the product of these two is kept constant to a fix expected value (EV = 0.16).

The network was able to perform greater than chance in choosing the best offer. As with our monkeys, errors (in which the network choice is the gamble with lower expected value) were most common when the two-presented gambles were close in value (Figure 2A, Strait et al., 2014, 2015). The performance of the network as an average over all instances converged to a final accuracy of 85% (variance across 300 instance of repeats was 11.56%, standard deviation = 33.99%), a quantity that approximates to the behavior of monkeys in similar tasks.

## Value is coded broadly in the network

In modular systems, each neuron's specific computational role is specialized and is determined by the function of the module to which it belongs. In distributed systems, by contrast, neurons tend to have broader and more flexible roles. These roles vary quantitatively but seldom qualitatively, and neurons may play multiple roles simultaneously. In our network, coding of all task variables was distributed.

Figure 2B shows that more than 80% of the neurons coded for all categories such as rewards (*blue line*), probabilities (*red line*), spatial position (*yellow line*), and choice (*violet line*). Figure 2C shows regression coefficients for the encoding of rewards, *r*_*L*_ (*blue line*), probabilities, *p*_L_ (*red line*), in each layer. All of these variables were coded ubiquitously and were not confined to a specific layer or to specialized neurons in a layer (Supplementary Figure 1). These results support the basic idea that an implementation of a decision process does not need to be composed of neurons that resemble distinct choice stages, such as a separate representation of probability and reward that is later combined for a decision.

**Figure 3:**
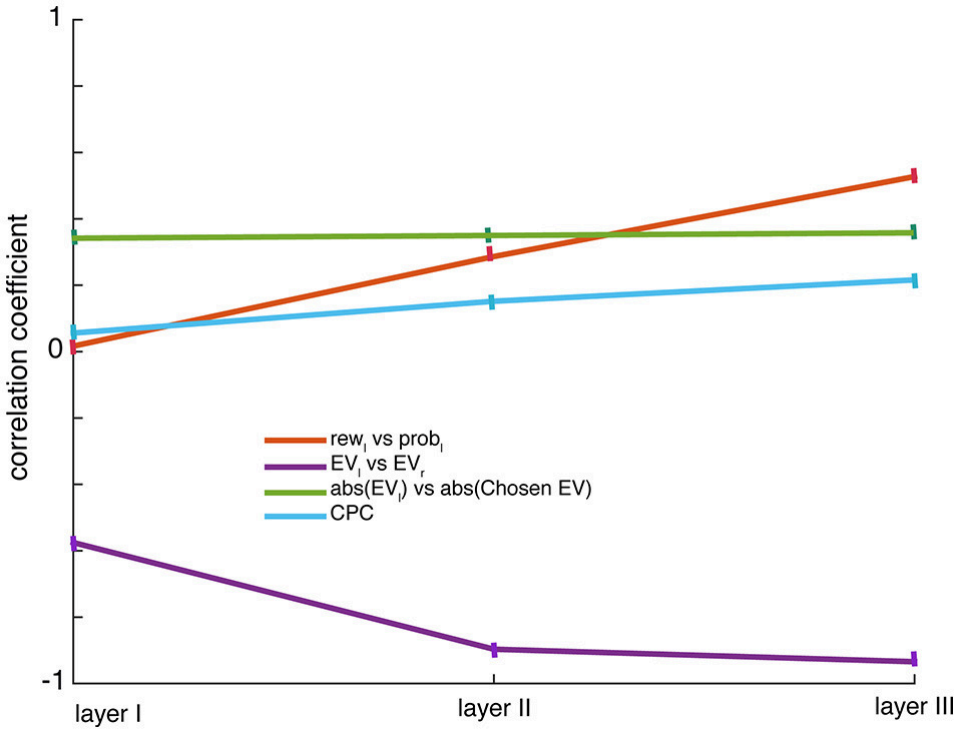
Correlations: The panel shows Pearson correlation coefficients measured for the scatter between regression coefficients (β) over the neuronal population for probabilities and rewards presented in left side: *rew*_l_ and *prob*_l_, the integration increases with layers; for expected values: *EV*_l_ and *EV*_r_, the anti-correlation for value tuning increases with layers; for abs(*EV*_l_) and abs(chosen value) there is a constant positive correlation between the offer value and the chosen value that doesn't change with layers. The regression coefficients are obtained from linear regression using a single regressor. Choice probability correlations (CPC) referring to a correlation between responses of neurons (variance in firing not accounted for by task variables such as offer value, offer probability, offer position) and choice increase as the level of hidden layer increases; error bars denote SEM.

Neurons that coded *p*_L_ were more likely to jointly encode *r*_L_ (for example in the third hidden layer: Pearson correlation, ρ = 0.53, *p* < 0.001, Figure 3*, red line*). Similar results were found for *r*_R_ and *p*_*R*_ (in the third hidden layer: ρ = 0.54, *p* < 0.001). This kind of multiple-value coding pattern was seen throughout the network (Supplementary Figure 2). The dependence of response, in the same way, on the two dimensions that determine value indicates that these neurons' responses correlated with integrated value. (The fact that correlations are substantially weaker than 1 suggests that value may be only partially integrated at this point). The positive relationship between coding for elements that contribute to value is a diagnostic feature of value coding in single neurons. The proportion of neurons encoding *EV*_L_ and *EV*_R_ increased in higher layers (Kruskal-Wallis, χ^2^ = 15.34, *p* < 0.05), suggesting that the conversation of disjunct inputs to values occurs gradually and smoothly rather than in one discrete layer. Overall, value coding was ubiquitous and there weren't distinct neurons coding only value.

### Neural parallels

The broad coding of value correlates mirrors neural results showing that reward signals and their expectancies can be found ubiquitously throughout the brain, from early sensory structures (Shuler and Bear, 2006; Serences, 2008) to premotor structures (Cisek and Kalaska, 2005; Cisek, 2007). Indeed, one recent neuroimaging study reported coding of reinforcement signals broadly throughout much of the brain (Vickery et al., 2011). Single neuron data collected throughout the prefrontal cortex and striatum shows value correlates ubiquitously in these areas as well (e.g., Wallis and Rich, 2011; Nogueira et al., 2017). Some work has focused on distinguishing ersatz value signals—mere value correlates—from true value representations (Wallis and Rich, 2011; O'Doherty, 2014). This work is motivated, in part, by the idea that multiple value representations would be redundant and pointless. In a modular system, they would be. Our model suggests an alternative idea, that ubiquitous value correlates may be real, and may be a signature of distributed choice processes.

It is not the case in our network model that any layer precedes or follows the computation of value—although layers do differ quantitatively in how abstract their value encoding is. Value encoding becomes more prevalent and more abstract (i.e., there is greater integration) with layer; later layers simply inherit and purify less abstract value encodings from previous layers. This finding is consistent with some results showing more abstract (less-componential or less-dimensional) tunings in the anterior cingulate cortex (ACC; presumably later in the sequence) and less abstract or more componential in the orbitofrontal cortex (OFC; presumably early in the sequence, Kennerley and Wallis, 2009; Kennerley et al., 2011; Blanchard et al., 2015a; Nogueira et al., 2017).

## Value information is not segregated from spatial information

We next considered the encoding of spatial information, and defined coding of offer side as the difference in responses to the same offer (i.e., probability/reward pair) when it appeared in position 1 (left, first two terms) vs. position 2 (right, third and fourth term). Note that because it is a neural network, the spatial terms are notional; what is important is the linkage between the first pair of input neurons and the first output neuron and the linkage between the second pair of input neurons and the second output neuron. We found that encoding of offer side and chosen side rose as layer level increased (Figure 2C*, violet and yellow lines respectively*). Thus, spatial information—from both the input (offer) and output (choice) end—is present throughout the network. More generally, information about the details of the stimulus—information that allows for identification of stimuli and specification of actions, is present throughout the network. Therefore, there are no pure abstract *value* units that solely code expected value (Pearson correlation between unsigned regression coefficients for *spatial position* and *EV*, ρ = 0.2, *p* < 0.001 suggesting their joint encoding).

It is notable that neurons do not consistently use the same code (left vs. right) for offer (Figure 2C*, violet line*) and choice (Figure 2C*, yellow line*). In other words, a left-preferring neuron for offers is no more or less likely to be left-preferring for choices. It appears then that our network does not develop a task invariant spatial code. In other words, space is not accounted for in any special way and does not serve as an anchor around which other signals are organized, but rather different spatial signals are mapped to units arbitrarily. One implication of the fact that our network lacks neurons with only a pure value code is that any downstream decoder will need to have information about the position of a gamble to be able to ascertain the meaning of a value response, at least from a single neuron. This feature is characteristic of distributed systems.

### Neural parallels

The question of whether spatial position can be decoded in reward regions of the brain is much debated (Feierstein et al., 2006; Roesch et al., 2006; Cai and Padoa-Schioppa, 2012; Strait et al., 2016). There is some evidence that the OFC and/or the ventromedial prefrontal cortex (vmPFC) may be a space-free region (Padoa-Schioppa and Assad, 2006; McNamee et al., 2013; Grattan and Glimcher, 2014; Rich and Wallis, 2016). Indeed, the purported lack of spatial information in OFC/vmPFC is one factor supporting the idea that they, not anterior cingulate cortex, are candidates for the core value regions of the cortex (Heilbronner and Hayden, 2016; Strait et al., 2016). However, the situation is clouded by other evidence pointing to spatial information in these regions, especially for chosen action (Feierstein et al., 2006; Seo and Lee, 2007; Furuyashiki et al., 2008; Tsujimoto et al., 2009; Sul et al., 2010; van Wingerden et al., 2010; Abe and Lee, 2011; Luk and Wallis, 2013; Rich and Wallis, 2014; Bryden and Roesch, 2015; McGinty et al., 2016; Strait et al., 2016), and also for the offer position (Strait et al., 2016).

If spatial information for offer and choice is contained throughout the brain's reward regions, our network makes a suggestion of why. The network gradually begins to build a representation of the forming action plan. Information about the plan is weak but detectable even in early layers. And information about the positions of offers (to the extent that it is irrelevant to choice) is weak but detectable even in later layers. From that perspective, then, there is no real categorical distinction between goods-based (input-based) and action-based (output-based) choices; there is just a gradual transformation from input to output domains. Although early layers may be more goods-based-like and later layers may be more action-based-like. From this perspective, then, goods-based and action-based choice is a false dichotomy, and value comparison among goods and action selection for a choice are two names for the same process (Cisek and Kalaska, 2010).

## Relevance of spatial selectivity results to rule encoding

Often times, we must deal flexibly with changing rules to make effective choices (Wallis et al., 2001; Yamada et al., 2010; Sleezer et al., 2016). For example, the decision about whether to answer or ignore a cellphone ringtone depends on whether it is one's own phone or a colleague's. The rule then is a modulatory factor that changes an input-output mapping. In our network, which models space in an abstract manner, space is just another rule. That is, if the positions of two gambles are switched, the action may be entirely different, even if the evaluation and comparison are the same. Indeed, if we wished to model rule instead of spatial position, our network would be identical, and its results would of course not change. For this reason, the spatial findings have some implications for the neuroscience of rule-based decisions in distributed networks as well. Specifically, they suggest that we should expect to find rule representations embedded within the same set of neurons that make choices.

### Neural parallels

The neuroscience of rule-based decision-making, like neuroeconomics, often proceeds from the assumption of modularity. Thus, it is often assumed that rules are stored in specialized brain regions that modulate activity in other disparate regions to implement rules (Miller and Cohen, 2001; Wallis et al., 2001; Wallis and Miller, 2003; Yamada et al., 2010). While the evidence for a modular organization of rule processing systems is strong, some recent results challenge this idea. Most notably, some results indicate that rule encoding is found in a wide number of structures. Most notably, results indicate that rule encoding can be observed in ostensibly core reward regions like OFC and ventral striatum, VS (Wallis et al., 2001; Floresco et al., 2006; Hayden et al., 2010; Bissonette and Roesch, 2015; Sleezer and Hayden, 2016; Sleezer et al., 2017).

## No common currency code

The overlap at the single neuron level between value and spatial coding challenges the idea that there is necessarily a pure value domain. In doing so, this finding raises a deeper question, of whether a single domain-independent value code is necessary. Such a code is sometimes called a common currency code because it allows for direct comparison of dissimilar goods (Landreth and Bickle, 2008; Chib et al., 2009; Padoa-Schioppa, 2011; Levy and Glimcher, 2012; Sescousse et al., 2015).

We reasoned that in if our network uses a common currency value system, then equally valued gambles would elicit the same responses regardless of what drove that valuation. Thus, a high-stakes low-probability and a low-stakes high-probability gamble that are equally preferred should produce the same responses. Formally, we say a common currency format is used if the response to offers A and B is the same when the value (as measured by preference indifference) of A and B are matched but their attributes (reward and probability) are not. Our network did not produce any neurons exhibiting common currency effect on analyzing all EV and all layers. Figure 2D presents few instances; it shows responses from example neurons in third layer for different combination of reward and probability components (*x labels*) yet with same expected value, 0.16. The results show that responses are distinct for various combinations of rewards and probabilities suggesting that there is no common currency coding.

Another basic assumption of a common currency code is that responses of neurons that use such a code will encode the value of offer and not its attributes (reward, probability). We showed above that integration rises with layer in the network and is greatest in the third layer. But does the network ever succeed in throwing away all information about components to create a pure value signal? It appears the answer is no: it was possible to decode both attributes independently from ensemble responses from any layer of the network. The network codes offer values (EV) for left and right positions very differently (Pearson correlation ρ = −0.58 in layer I, −0.9 in layer II and−0.93 in layer III, *p* < 0.001, Figure 3*, violet line*), while still differentially coding for their attributes—rewards (Pearson correlation between signed regression coefficients for *r*_L_ and *r*_R_, ρ = −0.42 in layer I, −0.71 in layer II and −0.79 in layer III, *p* < 0.001) and probabilities (Pearson correlation between signed regression coefficients for *p*_L_ and *p*_R_, ρ = −0.19 in layer I, −0.48 in layer II and −0.66 in layer III, *p* < 0.001) of offers. They show that offer's expected values *EV*, and their attributes, *r* and *p* can be decoded independently.

These results indicate that, whether or not we can say that the network uses a common currency code in any sense (this may be a philosophical question), it is not one that is observable at the level of the single unit by our definitions. Together with the above findings, these results challenge the idea that value must be “recognizably coded” (to use Fetz' term, Fetz, 1992) or be reified (to use the philosophical term) at the single unit level in a system that can make economic choices between gambles differing on multiple dimensions. On the contrary, they show that it is possible for even a simple system to make effective choices without solely computing and encoding value in its neurons. Value can be coded emergently—that is, it is not present in any units particularly, but observed as an output of the system as a whole.

### Neural parallels

A good deal of evidence supports the idea that neurons in a few brain regions use a common currency code for value, especially OFC (e.g., Padoa-Schioppa, 2011; Levy and Glimcher, 2012). We consider this evidence to be relatively strong, but anticipate that more sophisticated analysis methods and larger recordings in more complicated tasks will be needed to critically test these ideas. Some tentative new results have already challenged the dominant view of OFC as a source of common currency codes (McGinty et al., 2016; Wang and Hayden, 2017). Nonetheless, we regard the question as currently unresolved.

## No labeled lines for offers

Several prominent models of value comparison are *labeled line* models; that is, they imagine two discrete populations of neurons competing for control of dedicated comparison neurons (e.g., Chau et al., 2014; Rustichini and Padoa-Schioppa, 2015; Hunt et al., 2016). Labeled line model fits readily into a modular organizational scheme: the labeled lines neurons are pre-decisional; the comparison neurons are decisional, and their targets are post-decisional. They do not fit as naturally with a distributed model.

We did not see two distinct populations of neurons for the two offers in any layer of our network. Specifically, we found a positive relationship between coding strength (unsigned regression coefficient) for the two offers, a finding that cannot be reconciled with the idea of specialized populations for the two values (Pearson correlation, ρ = 0.08, *p* < 0.0001). Similar positive correlation between unsigned regression coefficients for offer and chosen value was found (ρ = 0.35, *p* < 0.05, Figure 3*, green line*). These findings suggest that offer and chosen value neurons do not constitute discrete sets of neurons (Figure 3).

### Neural parallels

It remains unclear whether neurons in the reward system have labeled line encodings. There is a small amount of evidence that neurons that encode the value of the two offers in a choice are the same, but alternate in which value they encode - a finding at odds with labeled line models (Rich and Wallis, 2014; Strait et al., 2014, 2015; Blanchard et al., 2015a; Azab and Hayden, 2017; Xie et al., 2017). These lines of work have not yet been reconciled, but the question is empirical. The contribution of our model, however, is to show that a labeled line system is not theoretically necessary, or one that a distributed system will necessarily develop through simple learning rules.

## Comparison of values

We found a significant negative correlation between the signed coding parameters for all layers (for value in the first layer: Pearson correlation, ρ = −0.58, *p* < 0.001). That is, to the extent that a neuron encodes the value of one offer it (stochastically speaking) encodes the value of the other offer with reversed tuning. Thus, no neuron is solely dedicated to a particular offer, consistent with the lack of labeled line coding (see previous section). We have previously reported such an effect in vmPFC, VS, and dorsal ACC, and have attributed this effect to a comparison process (Strait et al., 2014, 2015; Azab and Hayden, 2016). Indeed, in our network, this negative correlation, which was distributed across layers, is the mechanism by which comparison and thus choice occurs.

The anti-correlation between the value tuning for the two offers rose with layer (Figure 3, *violet line*; Pearson correlation, ρ = −0.93, *p* < 0.001), suggesting that all layers contribute to comparison but that responses of later layers more closely resemble a value difference. In other words, early layers initiate the process of value comparison and later layers strengthen it by aggregating signals from earlier layers. Thus, a consensus between early and late layers can be achieved directly in a feed-forward manner. This does not mean that the “true” site of comparison is the last layer, and the earlier ones are simply modulators of the comparison. We could equally say that the comparison occurs in rough form in the first layer, and is refined in subsequent layers. More simply, we could say that the choice is distributed.

Choice probability correlation (CPC) is a term that refers to a correlation between responses of neurons (variance in firing not accounted for by task variables) and choice. It can be computed by analyzing choice correlations for our network's responses after regressing out task input variables such as reward, probability and spatial location of the two gambles. We found significant but weak choice correlations in neurons throughout the system (Kruskal-wallis, χ^2^ = 21.643, *p* < 0.05). The size of correlations rose with layer (Figure 3*, blue line*).

## Lesions lead to graceful degradation of function

Studies on lesions in the decision making circuitry don't provide consistent results for behavioral impairment (Harlow, 1869; Damasio and Damasio, 1990; Bechara et al., 2000); as there is evidence for little or no change in decision making behavior even after surgical removal of entire frontal lobe (Busch et al., 2017). Several theories point toward the presence of underlying distributed networks, where functions are not localized, for reconciling with the results of lesion studies (e.g., Plaut, 1995). Specifically, many studies relate to *graceful degradation* property of distributive nets to explain lesion effects (Farah, 1994; Plaut, 1995; Wilson et al., 2010).

**Figure 4:**
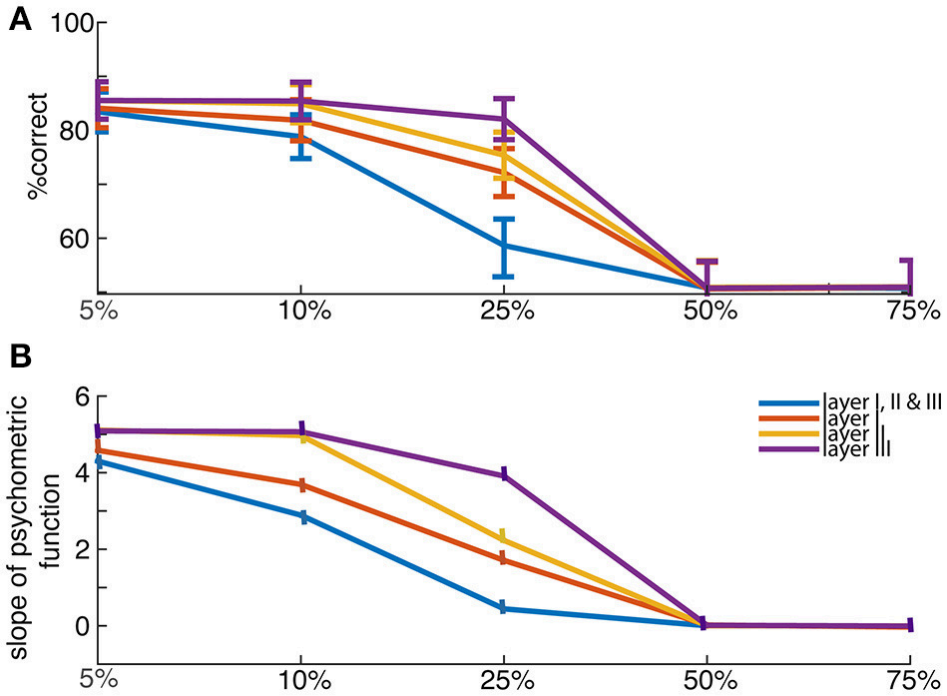
Lesion effects **(A)** Network's performance in choosing the best offer when averaged over trials, and **(B)** the crossing slopes of sigmoid curves (psychometric function as in Figure 2A, for percent choosing the offer presented in left as a function of value difference between left and right offers) fitting those percentages, are shown for lesioned networks—with percent lesions 5, 10, 25, 50, and 75% (for layers I, or II, or III, or all of the three layers, as mentioned in legend). The psychometric curves were fitted using a hyperbolic tangent function to show network's average performance.

Graceful degradation of function is a standard property of neural networks (Arbib, 2003; Haykin and Network, 2004). We explored the effects of network lesions in sizes of 10–75% per layer. Lesions were generally not catastrophic (a benefit of distributed processing networks). They were modeled by nullifying the responses of certain proportion of neurons that are randomly selected in a layer. It led to choice deficits that were stronger for more difficult choices. Particularly, smaller lesions (25% size) were less destructive than medium (50% size) and large (75% size) lesions for all layers, as expected (Figure 4).

## Conclusions

The distributed implementation of economic choice may be less familiar or less intuitive than a modular one, but it should not be. Familiar distributed decision-making processes include how bees, ants, and other social insects choose hive sites, how fashion trends become popular, and how prices are set in capitalist economies (Bartels, 1988; Franks et al., 2002; Cohen et al., 2008; Seeley, 2010). These are processes in which there is no central decision-maker; instead, the choice is the result of simple repeated interactions among agents that all have a limited and often noisy view of the whole (Mitchell, 2009). In this perspective, we focus on implementing a classic model of neural networks to delineate some properties of distributed networks as put forth earlier by several scholars (Cisek and Kalaska, 2010; Cisek, 2012; Pearson et al., 2014; Eisenreich et al., 2017; Hunt and Hayden, 2017), and compare them to neural data, conceptually, to quantify how they can adjudicate between distributed and modular models of economic choice. We happened to choose a neural network to implement it because it's a well-understood system. In our model, we find the neurons implementing evaluations, comparisons, and choice, ubiquitously; qualitative differences arise between layers. Some results on ubiquitous presence of comparison between offers, and choices (in form of CPCs) are shown in the last section of the results- “comparison of values.” We also performed a simple modification to the network, by adding one another layer at the end consisting of a single binary output neuron, directly reading the choice (Supplementary Figure 3). This modification didn't alter any of our results qualitatively, suggesting the network didn't wait till the end to perform comparisons and choices, substantiating they are ubiquitously spread throughout the network.

One major limitation of our network is that it is designed to perform only a single task. It is possible that a network designed to be more flexible to tackle complex variety of tasks will naturally create more modularity in its organization (Yang et al., 2017). However, our brief consideration of space and rules in the brain suggest that such modularity is not necessarily observed (also see Hunt and Hayden, 2017). Another limitation would be the simplicity of computational neural network we chose to perform the distributed computations. Perhaps, more complex networks can be thought to model the brain functions closely, but nevertheless we seek to show that basic properties of simple distributed networks could explain the principles of neuroeconomic choice as observed from our past electrophysiological data. We made the decision to focus on linear correlations primarily because neural studies use that measure; they thus allow for the most direct comparison with existing neural data and thus familiar to most readers. This is a possible limitation of the current study. However, the main idea of the study, distributed coding of the neural network, can be claimed irrespective of the linearity of its statistical methods. We can notice them from Figure 2D, where we test the encoding of expected value, EV, in all neurons, and the results show none to encode purely EV regardless of other variables. Therefore, the neurons of our network don't exhibit common currency.

One property that distinguishes many distributed systems from many modular systems is the relative self-similarity of the system across disparate regions. Another (sometimes) distinguishing property is the relatively direct relationship between functions of neurons and functions of the whole (Mitchell, 2009). These features have direct implications for the interpretation of neural data. If the brain is highly modular, like a microprocessor, then responses of single units can be nearly useless and often highly misleading when making inferences about the whole (Jonas and Kording, 2017). However, to the extent that the brain works in a distributed manner, responses of units can be diagnostic about the properties of the system, making unit physiology useful. Again, this is not to say that the brain is highly distributed, or that strong and well thought-out theories are not helpful. But, the possibility of a distributed brain may be one reason for optimism when faced with limited measures of brain activity.

One reason given to favor a highly modular approach over a distributed one is that abandoning modularity means giving up answering any important questions like what distinct roles given regions and neurons play. Supporting a distributed view, then, is seen as pessimistic, giving into a neural nihilism. We do not agree. Instead, we think that while adopting a distributed approach does demote some questions, it promotes a new and different set of questions. Most fundamentally, how can very simple computational neurons be arranged so that they produce flexible and accurate choices? And what simple learning rules can these neurons follow that allow them to adapt to changing circumstances in some cases, and yet to store important information for decades in others?

Why factors, then, might make us favor a distributed system to a modular one—or a hybrid to a purely modular one? One fundamental motivating factor behind distributed systems is their naturalness—that is, they are more naturally evolved. Brains are created over millions of years to solve particular problems (Fetz, 1992; Cisek and Kalaska, 2010; Cisek, 2012; Hayden, 2015, 2018; Jonas and Kording, 2017). As such, they are limited in the pathways they can take toward certain organizations. At the same time, our behaviors can be consistent with multiple brain organizations—they may be multiply realizable. Several features of our network seem consistent with unplanned, self-organized systems. For example, the lack of specific links between neurons and functions (Churchland et al., 2012; Rigotti et al., 2013; Blanchard and Hayden, 2018). Likewise, the ability of single regions to perform multiple functions simultaneously is consistent with a bottom-up function (Cisek and Kalaska, 2005; Hayden and Gallant, 2013; Luk and Wallis, 2013; Pearson et al., 2014; McGinty et al., 2016; Nogueira et al., 2017). We have argued that taking the perspective based on the constraints imposed by evolution can shed new light onto our understanding behavior (Blanchard and Hayden, 2015). We suspect the same is true for neural responses as well.

The goal of the present study is to delineate some basic principles of economic choice in distributed systems. Because our ultimate goal is to understand brain function, and because neural networks are a particularly tractable distributed system, we used them as our model here. Our model was not designed with a goal of biological realism; nonetheless, we think that some of the similarities between responses in our network and those observed in neurons are suggestive, and support the idea that the brain's choice mechanisms may be more distributed than is sometimes thought. This does not mean that the brain is a fully distributed chooser however. Indeed, the evidence for modular function is copious (e.g., Rushworth et al., 2011). It is not, however, unambiguous. Some methods (lesion studies, neuroimaging) are more well suited for detecting modular functions and others (unit physiology) are relatively more well suited for detecting distributed ones. Thus, the methods and techniques used by various laboratories could bias their inferences on brain organization, As such, the portrait of choice derived from these methods are different, sometimes strikingly so. Ultimately, however, we suspect that the truth lies somewhere in the middle.

Given the likely possibility that the truth lies in between, the question of why the field has focused on modular models is an interesting one. We suspect that part of the reason is that such models are more intuitive and familiar, especially to scholars more familiar with computers than with ant colonies (Eisenreich et al., 2017). And another reason is that the most widely used method to study economic choice, neuroimaging in humans, is more adept at assigning functions to regions than it is as detecting distributed computations. We also suspect that in the future, models designed with more biological realism in mind could help sort out the differences between different methods, such as fMRI, lesion studies, and unit physiology.

## Author contributions

PB, RM-B, and BH: Contributed to conceiving of idea, designing of methods, analysis of results, and manuscript writing.

### Conflict of interest statement

The authors declare that the research was conducted in the absence of any commercial or financial relationships that could be construed as a potential conflict of interest.

## Supplementary material

Supplementary Figure 1 Encoding of option dimensions. **(A)** prob_l_ **(B)** rew_l_ **(C)** offer side **(D)** chosen side layers 1, 2, and 3 (three subpanels). The forth and fifth subpanels present summary statistics for all three layers- variance of distribution and the proportion of nodes of encoding variables in **(A–D)**, and error bars show SEM.

Supplementary Figure 2 Correlations. Between **(A)** unsigned regression coefficients for probabilities and rewards presented in a specific position, here, left side, Abs(prob_l_) and abs(rew_l_), **(B)** signed regression coefficients for probabilities and rewards presented in left side, rew_l_ and prob_l_, **(C)** unsigned regression coefficients for expected values, abs(EV_l_) and abs(EV_r_), **(D)** signed regression coefficients for expected values, EV_l_ and EV_r_, **(E)** EV_l_ and chosen value; and three subpanels in **(A–E)** for all three layers. Each subpanel also presents its data's Pearson correlation coefficient (rho represented as “r”) and *p*-value (*p*).

Supplementary Figure 3 Choices, comparisons and evaluations are ubiquitously spread throughout the network. This extended network consists of an additional layer at the end with a single output neuron. We find their results to be qualitatively similar to our earlier network dealt in this manuscript. **(A–C)** Show the basic behavior and correlations found in the extended network. **(A)** Shows the accuracy of choice through time; **(B)** shows the percent choosing of offer presented in the left as a function of value difference between left and right offers; **(C)** shows correlations similar to Supplementary Figure 2. Simulation results for the extended network are run for total instances of 10, and trials of 15,000. The responses of neurons in the final 1,000 trials portraying stationary performance through a plateau are used for analysis. The architecture of the extended network consists of initial input layer of size 4 (similar in properties to our earlier network), hidden layer 1 of size 50 neurons, hidden layer 2 of size 50 neurons, hidden layer 3 of size 50 neurons, hidden layer 4 of size 2 neurons, output layer of size 1 neuron. Learning rates are set to 0.01. Activation functions are designed with same properties as our previous network, and the newly added output layer has a *tanh* activation function of slope 10. The output neuron directly reads choice of offer 1 (offer 2) depending on its activity being greater (lesser) than zero. The back-propagation training uses error function constructed as a difference between the choice read by the network and the desired (accurate) choice.
