# Supplementary figures and images for: Using a Simple Neural Network to Delineate Some Principles of Distributed Economic Choice
Source: Front Comput Neurosci. 2018 Mar 28;12:22. doi: 10.3389/fncom.2018.00022 (PMC5882864; doi:10.3389/fncom.2018.00022)

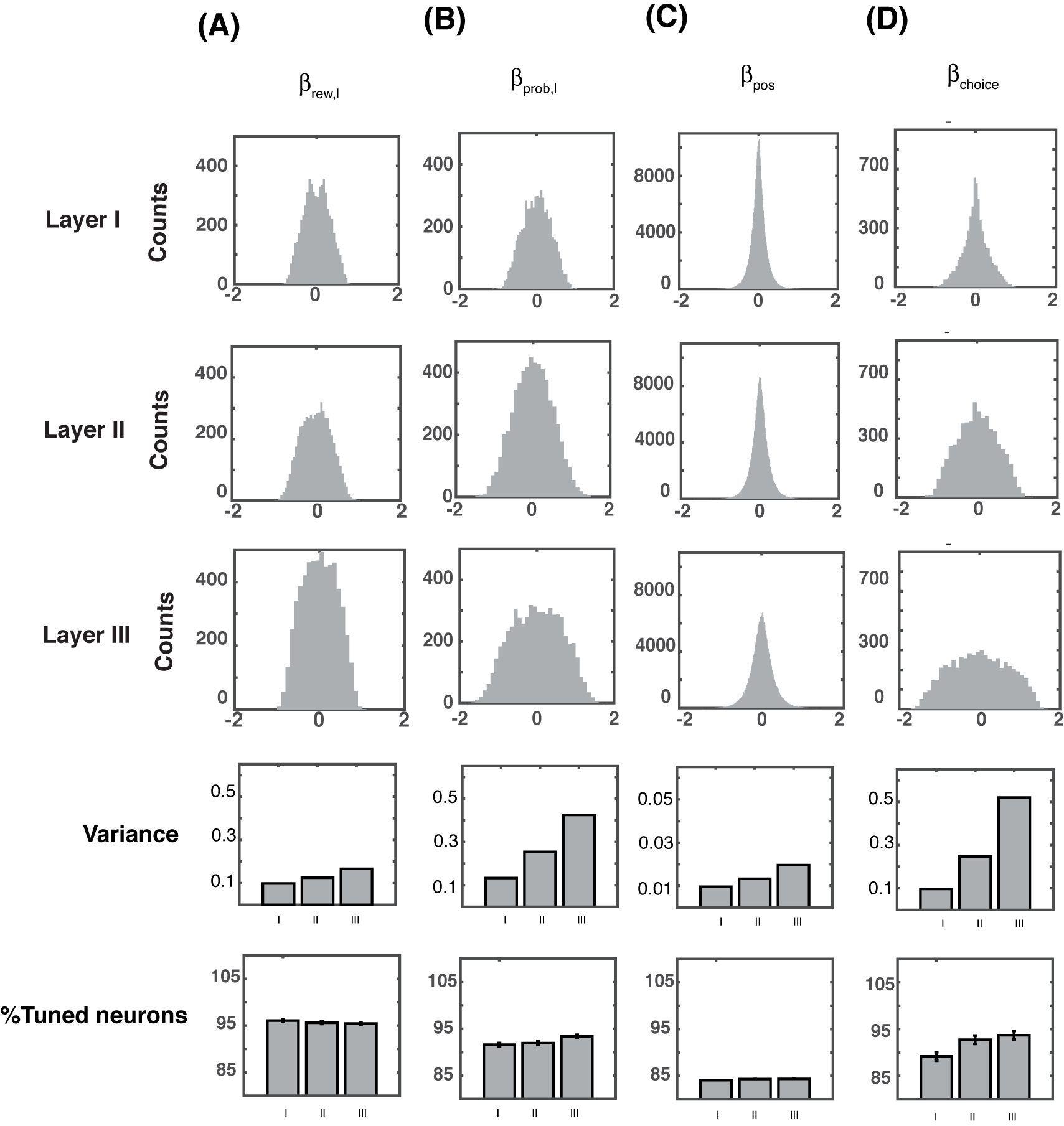

Supplement: Supplementary Figure 1 — Encoding of option dimensions. (A) probl (B) rewl (C) offer side (D) chosen side layers 1, 2, and 3 (three subpanels). The forth and fifth subpanels present summary statistics for all three layers- variance of distribution and the proportion of nodes of encoding variables in (A–D), and error bars show SEM. [file Image1.tif]

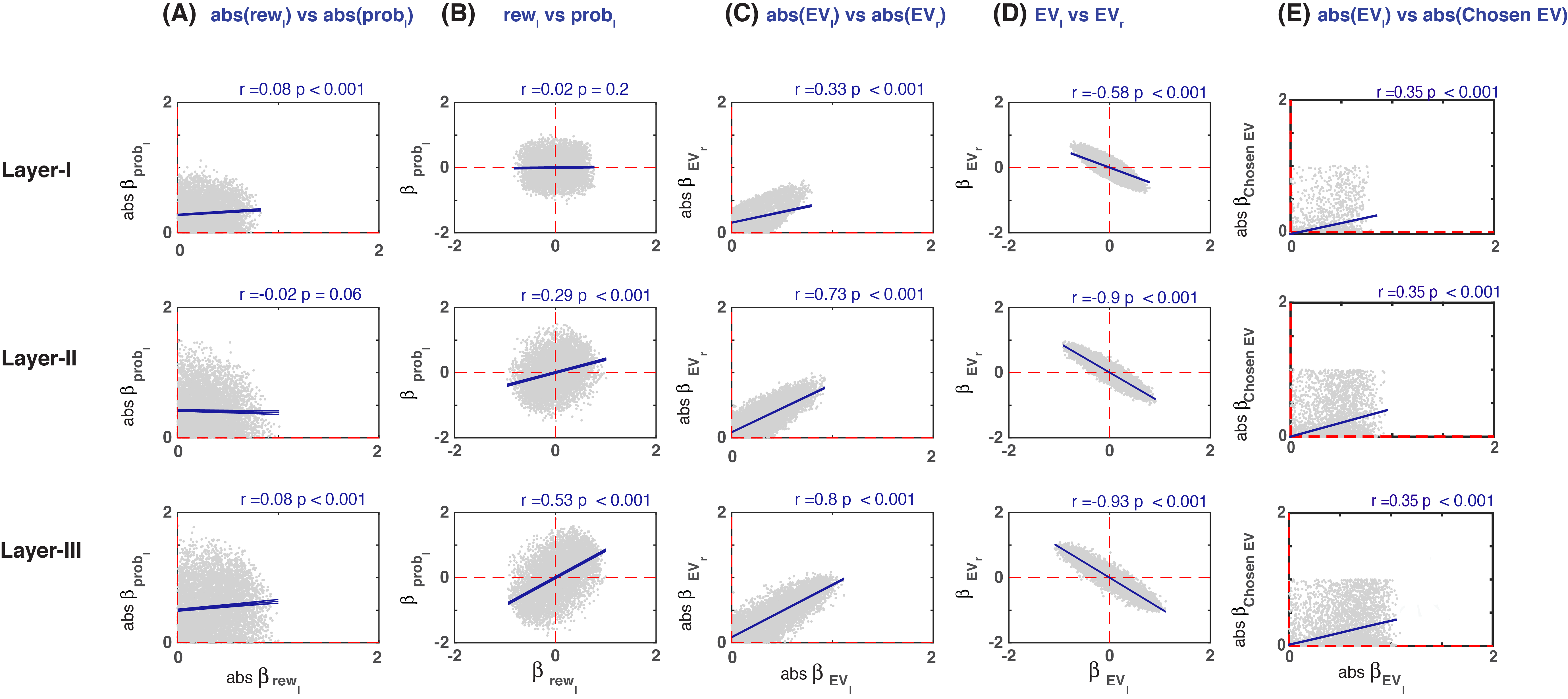

Supplement: Supplementary Figure 2 — Correlations. Between (A) unsigned regression coefficients for probabilities and rewards presented in a specific position, here, left side, Abs(probl) and abs(rewl), (B) signed regression coefficients for probabilities and rewards presented in left side, rewl and probl, (C) unsigned regression coefficients for expected values, abs(EVl) and abs(EVr), (D) signed regression coefficients for expected values, EVl and EVr, (E) EVl and chosen value; and three subpanels in (A–E) for all three layers. Each subpanel also presents its data's Pearson correlation coefficient (rho represented as “r”) and p-value (p). [file Image2.PNG]

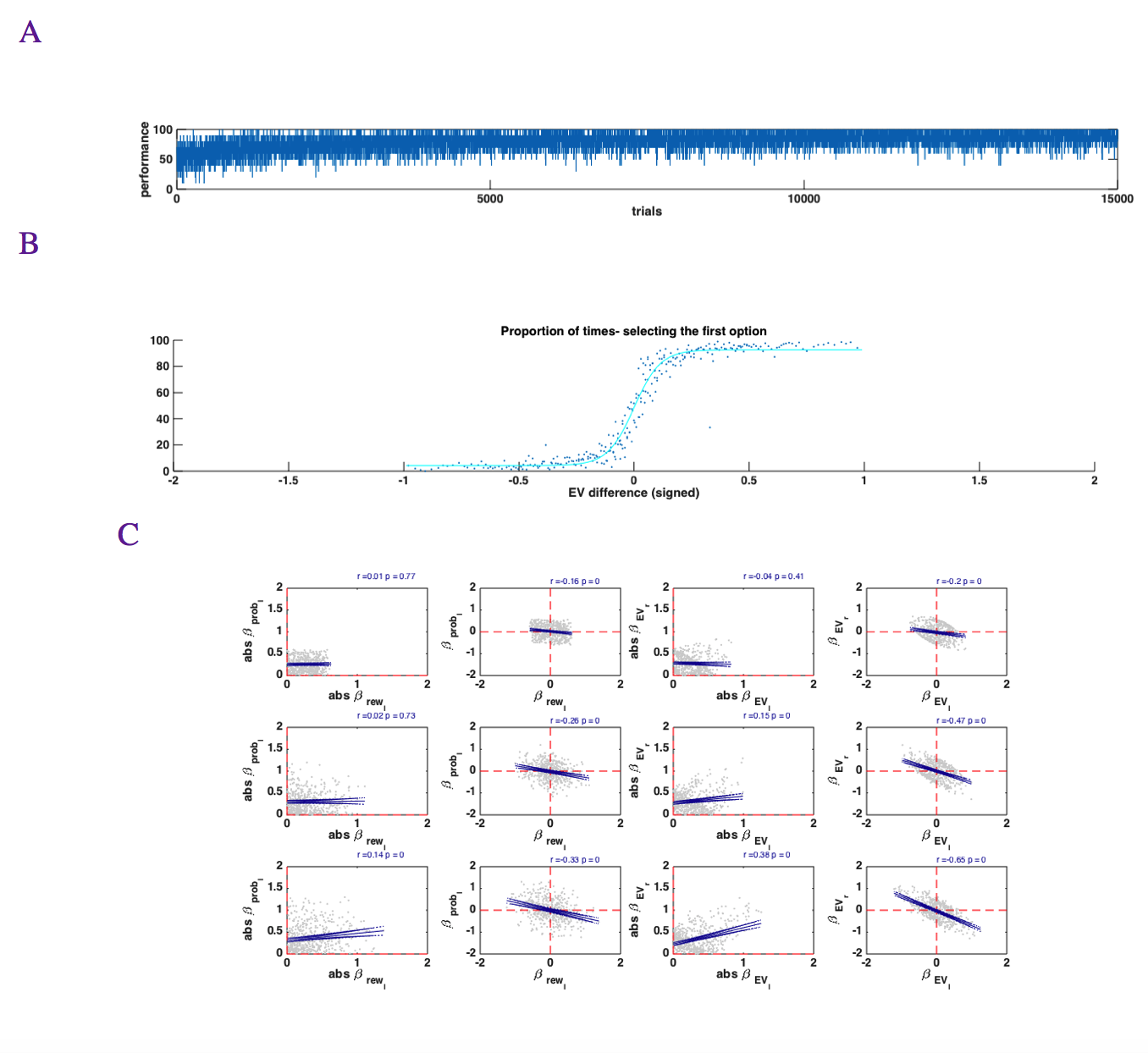

Supplement: Supplementary Figure 3 — Choices, comparisons and evaluations are ubiquitously spread throughout the network. This extended network consists of an additional layer at the end with a single output neuron. We find their results to be qualitatively similar to our earlier network dealt in this manuscript. (A–C) Show the basic behavior and correlations found in the extended network. (A) Shows the accuracy of choice through time; (B) shows the percent choosing of offer presented in the left as a function of value difference between left and right offers; (C) shows correlations similar to Supplementary Figure 2. Simulation results for the extended network are run for total instances of 10, and trials of 15,000. The responses of neurons in the final 1,000 trials portraying stationary performance through a plateau are used for analysis. The architecture of the extended network consists of initial input layer of size 4 (similar in properties to our earlier network), hidden layer 1 of size 50 neurons, hidden layer 2 of size 50 neurons, hidden layer 3 of size 50 neurons, hidden layer 4 of size 2 neurons, output layer of size 1 neuron. Learning rates are set to 0.01. Activation functions are designed with same properties as our previous network, and the newly added output layer has a tanh activation function of slope 10. The output neuron directly reads choice of offer 1 (offer 2) depending on its activity being greater (lesser) than zero. The back-propagation training uses error function constructed as a difference between the choice read by the network and the desired (accurate) choice. [file Image3.PNG]
